# Family Eating Habits and Dietary Quality of Spanish Children and Adolescents: The PASOS Study

**DOI:** 10.3390/nu18071038

**Published:** 2026-03-25

**Authors:** Marina Ródenas-Munar, Silvia García, Santiago F. Gómez, Marcela González-Gross, Julia Wärnberg, Narcis Gusi, Susana Aznar, Elena Marín-Cascales, Miguel Ángel González-Valeiro, Susana Pulgar, Inmaculada Bautista, Maddi Osés, Luis Cereijo, Adela Martín-Oliveros, Montse Fitó, Paula Berruezo, Augusto G. Zapico, Juan Carlos Benavente-Marín, Jesús Sánchez Gomez, Evelyn Martin-Moraleda, Pedro E. Alcaraz, Marta Sevilla-Sanchez, Estefanía Herrera-Ramos, Idoia Labayen, Luis Carmona-Rosado, Ana Mateos-Lardiés, Helmut Schröder, Cristina Bouzas, Josep A. Tur

**Affiliations:** 1 Research Group on Community Nutrition and Oxidative Stress, University of Balearic Islands-IUNICS, 07122 Palma de Mallorca, Spain; 2 Centro de Investigación Biomédica en Red Fisiopatología de la Obesidad y la Nutrición (CIBEROBN), Institute of Health Carlos III, 28029 Madrid, Spain; 3 Health Research Institute of Balearic Islands (IdISBa), 07120 Palma de Mallorca, Spain; 4 Gasol Foundation Europe, 08830 Sant Boi de Llobregat, Spain; 5 CIBER de Epidemiología y Salud Pública (CIBERESP), Instituto de Salud Carlos III, 28029 Madrid, Spain; 6 Cardiovascular Risk and Nutrition Research Group (CARIN), Hospital del Mar Institute for Medical Research, 08003 Barcelona, Spain; 7 Nursing and Physiotherapy Department, University of Lleida, 25198 Lleida, Spain; 8 ImFINE Research Group, Department of Health and Human Performance, Universidad Politécnica de Madrid, 28040 Madrid, Spain; 9 EpiPHAAN Research Group, Faculty of Health Sciences, Universidad de Málaga-Instituto de Investigación Biomédica de Málaga (IBIMA Bionand), 28590 Málaga, Spain; 10 Physical Activity and Quality of Life Research Group (AFYCAV), Faculty of Sport Sciences, University of Extremadura, 10003 Cáceres, Spain; 11 Instituto de Investigación e Innovación en el Deporte (INIDE), 10003 Cáceres, Spain; 12 PAFS Research Group, Faculty of Sports Sciences, University of Castilla-La Mancha-Toledo Campus, 45071 Toledo, Spain; 13 UCAM Research Center for High Performance Sport, Universidad Católica de Murcia, 30107 Murcia, Spain; 14 Faculty of Sport, Universidad Católica de Murcia, 30107 Murcia, Spain; 15 Faculty of Sports Sciences and Physical Education, Universidade da Coruña, 15071 A Coruña, Spain; miguel.gonzalez.valeiro@udc.es (M.Á.G.-V.);; 16 Regional Unit of Sports Medicine of Principado de Asturias, Fundación Deportiva Municipal de Avilés, 33401 Avilés, Spain; 17 Research Institute of Biomedical and Health Sciences (IUIBS), University of Las Palmas de Gran Canaria, 35016 Las Palmas, Spain; 18 Preventive Medicine Service, Centro Hospitalario Universitario Insular Materno Infantil (CHUIMI), Canarian Health Service, 35016 Las Palmas, Spain; 19 ELIKOS Group, Institute for Sustainability and Food Chain Innovation (IS-FOOD), Department of Health Sciences, Public University of Navarre, 31006 Pamplona, Spain; 20 Public Health and Epidemiology Research Group, School of Medicine and Health Sciences, Universidad de Alcalá, 28871 Alcalá de Henares, Spain; 21 Sociedad Española de Farmacia Clínica, Familiar y Comunitaria (SEFAC), 28045 Madrid, Spain

**Keywords:** parents, children, adolescents, quality of diet, childhood obesity, sociodemographic factors

## Abstract

**Background**: Childhood nutrition is essential for development and disease prevention. Parental dietary practices and sociodemographic factors shape children’s eating habits. **Objective**: To assess the association between parental diet quality, children’s diet, and nutritional status, as well as the influence of caregiver sociodemographic factors. **Design**: Cross-sectional analyses were conducted using data from two waves of the PASOS study (2019–2020 and 2022–2023), which are nationally representative multicentre observational surveys. The analyses were restricted to participants with complete information on parental diet quality, children’s diet quality, and relevant covariates. **Methods**: Participants aged 8–16 years from the PASOS 2019–2020 (*n* = 1028) and 2022–2023 (*n* = 572) studies were included. Caregivers provided sociodemographic information and completed the Short Diet Quality Screener (SDQS), a validated questionnaire to assess parental diet quality. Children’s diet quality was assessed using the validated KIDMED index. Based on parental SDQS scores, participants were classified into low (≤50th percentile) or adequate/high (>50th percentile) diet quality groups. Associations were analysed using logistic regression and Pearson correlation. **Results**: Higher parental diet quality was consistently associated with greater adherence to the Mediterranean diet and higher consumption of fruit, breakfast cereals, and fish among children in both study waves. Children whose caregivers had better diet quality also showed a lower prevalence of abdominal obesity. Parental diet quality was positively associated with children’s diet quality and inversely related to several adiposity indicators, although associations with anthropometric measures were generally weak. **Conclusions**: Family-based approaches are essential for improving diet quality and preventing childhood obesity.

## 1. Introduction

Nutrition during childhood plays a fundamental role in growth, development, and the prevention of chronic diseases throughout life. In this context, the family represents the primary environment in which eating habits are formed, as parents or caregivers directly influence children’s dietary choices through behavioural modelling, the availability of foods within the home, and the structure of family meals [1]. A recent systematic review examining prospective associations between parental feeding practices and children’s fruit and vegetable consumption highlights the importance of structured feeding strategies within the home environment. The review found that practices such as parental modelling of healthy eating, nutrition education, and the availability of fruits and vegetables were consistently associated with higher consumption of these foods among young children, while more controlling practices showed weaker or inconsistent associations with healthy intake patterns [2]. Similarly, parental feeding practices that promote encouragement and autonomy while avoiding excessive pressure around food have been associated with healthier dietary patterns during childhood [3]. Importantly, eating habits acquired during childhood tend to persist into adulthood and may increase the risk of obesity and related chronic diseases when unhealthy patterns are established early in life [4]. Parents’ feeding practices can therefore play a significant role in shaping children’s dietary behaviours, regardless of demographic factors [5].

Childhood obesity remains one of the most important global public health concerns due to its high prevalence and long-term health consequences. In Europe, approximately 11% of children aged 7 to 9 years are affected by obesity, while in Spain, 33.4% of children and adolescents aged 8 to 16 years are overweight, with a prevalence of obesity of 11.8% and more than 20% presenting abdominal obesity [6,7]. These figures highlight the magnitude of the problem and its association with increased risk of metabolic disorders and cardiovascular diseases from early ages [6,7]. Poor nutritional quality of food during childhood has been associated with a higher risk of obesity and adverse metabolic health outcomes, reinforcing the need to promote healthy eating environments early in life [8]. Current reviews have also identified modifiable determinants of childhood obesity and lifestyle-related behaviours in children, highlighting the role of family-related factors, including parental dietary habits [9].

Within the family context, the quality of parental diet and feeding practices is influenced not only by individual preferences but also by a range of sociodemographic determinants. Caregivers’ educational level, weight status, health behaviours, and perceived well-being have been associated with variations in children’s dietary quality and eating behaviours [10]. In addition, the socioeconomic context in which families live, including parental education and employment conditions, may influence access to healthy foods and opportunities to adopt healthier lifestyles [11]. A recent systematic review examining sugar-related food parenting practices and feeding styles further emphasises the importance of these family dynamics. The review found that highly controlling practices—such as restricting or using sugary foods as rewards—were frequently associated with the development of unhealthy dietary behaviours and increased preference for sugar-rich foods over time. In contrast, parenting approaches that emphasise structure, balance, and healthy food environments were associated with more favourable long-term dietary behaviours in children and adolescents [11].

Despite the growing body of evidence highlighting the importance of the family environment in child nutrition, important gaps remain in the literature. Many previous studies have focused primarily on specific parental feeding practices rather than examining overall parental diet quality. Furthermore, parental dietary patterns, children’s diet quality, and anthropometric outcomes are often studied separately, limiting a comprehensive understanding of how these factors interact. In addition, much of the available evidence is based on single-time-point analyses or geographically limited samples, which restricts the ability to evaluate whether these associations remain consistent across populations and over time [12].

Addressing these gaps is particularly relevant given the persistently high prevalence of childhood overweight and obesity and the increasing recognition that family-level determinants play a central role in shaping children’s health behaviours. Recent evidence suggests that parental feeding practices and the family environment are closely associated with children’s dietary behaviours and obesity-related outcomes, highlighting the importance of family-based prevention strategies [5,13]. In this context, examining parental diet quality alongside children’s dietary patterns and nutritional status may provide a more comprehensive understanding of family influences on childhood health.

Therefore, the present study aims to assess the association between parental diet quality, children’s diet quality, and nutritional status, as well as the influence of caregiver sociodemographic factors, using data from two waves of the nationally representative PASOS study in Spanish children and adolescents.

## 2. Methods

### 2.1. Study Design

This study aimed to assess the association between parental diet quality, children’s diet quality, and nutritional status, as well as the influence of caregiver sociodemographic factors. To address this objective, a secondary analysis was conducted using data from two cross-sectional waves of the Physical Activity, Sedentary Lifestyle and Obesity in Spanish Youth (PASOS) study. Specifically, data from the PASOS surveys conducted in 2019–2020 and 2022–2023 were analysed. Both waves were designed as observational, multicentre studies with national scope. A detailed description of the design, sampling procedures, and data collection methods of the PASOS project has been reported elsewhere [14].

### 2.2. Participants, Recruitment, and Ethics

The first edition of the Physical Activity, Sedentary Lifestyle and Obesity in Spanish Youth (PASOS 2019–2020) project was conducted between March 2019 and February 2020. The study population consisted of children and adolescents aged 8–16 years residing in Spain and their parents. The original PASOS sample included 3802 participants recruited from 247 educational centres distributed proportionally across the 17 autonomous communities, providing a nationally representative sample.

The second edition of the study (PASOS 2022–2023) was carried out between March 2022 and June 2023 and included 3176 participants recruited from 245 educational centres across Spain using the same methodology and sampling strategy.

For the purposes of the present analyses, the analytical sample was restricted to participants with complete information on parental diet quality, children’s diet quality, anthropometric measures, and relevant covariates. After excluding participants with missing data in these variables, the final analytical sample consisted of 1028 participants from the 2019–2020 wave and 572 participants from the 2022–2023 wave. Some caregiver variables contained missing values due to incomplete questionnaire responses; therefore, the number of observations may vary slightly across variables in the descriptive tables. Differences in sample size across variables reflect variable-specific missing data and are consistent with the complete-case approach applied in each analysis. Participants included in the analytical sample did not differ substantially from the original PASOS sample in terms of age and sex distribution. Although the original PASOS study was designed to provide a nationally representative sample, restricting the analysis to participants with complete data may limit the strict representativeness of the analytical sample.

The same methodology and inclusion/exclusion criteria were followed in both editions. Students with intellectual disabilities that prevented them from responding to the questionnaires were excluded. Each case was evaluated with the teacher and parents or legal guardians prior to exclusion. Informed consent was obtained from parents or legal guardians, as well as informed assent from participants included in the study.

The PASOS project was carried out in accordance with internationally recognised ethical principles for research involving human subjects. The study protocol was evaluated and approved by the Ethics Committee of the Fundació Sant Joan de Déu (Barcelona, Spain) in (code PIC-179-18; approved on 17 December 2018). The study was also registered in 2019 in an international clinical trial registry, the International Standard Randomised Controlled Trial Number (ISRCTN), under the identifier ISRCTN34251612; accessed on 8 August 2019 (https://doi.org/10.1186/ISRCTN34251612).

### 2.3. Data Collection

Data were collected using self-reported questionnaires administered to children and adolescents via an online platform, with the support of researchers present at the educational centres. Anthropometric measurements (weight, height, and waist circumference) were then taken following a standardised protocol. Parents also completed self-reported questionnaires in digital format at home.

Validated questionnaires were used to obtain information on participants’ lifestyles (physical activity, sedentary behaviour, sleep, diet, and emotional well-being), as well as socioeconomic factors (parents’ educational level and occupation) and environmental factors. All data were collected on site at the selected schools by trained field researchers, who received prior face-to-face training from the Gasol Foundation in order to minimise inter-observer variability. Anthropometric measurements were performed according to the standardised protocol of the World Health Organisation (WHO) [15].

### 2.4. Dietary Habits

The Short Diet Quality Screener (SDQS) questionnaire, validated in the Spanish population, was used to analyse the quality of the parents’ diet [16]. It consists of 18 items on the frequency of consumption of the main food groups. This questionnaire was administered independently to both parents, identified as adult 1 and adult 2. Adult 1 was defined as the person responsible for the primary care of the child, and only their responses were considered for the analyses in this study. The total sample for each edition was divided into two groups based on the median total score of the questionnaire, used as a grouping variable. Scores equal to or below the 50th percentile (39 points) were classified as indicative of a low or inadequate diet quality, while scores above the 50th percentile were considered representative of adequate or high diet quality. The median was used as the cut-off point to ensure balanced group sizes and maintain adequate statistical power for comparisons between groups. Additionally, sensitivity analyses were conducted to assess the robustness of the main findings using the continuous SDQS score and alternative categorisations based on tertiles, focusing on the association with abdominal obesity.

The eating habits or diet quality of children and adolescents were assessed using the KIDMED index, a validated tool for evaluating adherence to the Mediterranean diet in children and adolescents and previously validated in the Spanish population [17,18]. This questionnaire consists of 16 items assessing the consumption of specific foods and dietary habits, with dichotomous (yes/no) responses. Items reflecting positive aspects of the Mediterranean diet are scored +1, whereas items with a negative connotation are scored −1, resulting in a total score ranging from −4 to 12. According to the original classification proposed for the KIDMED index, participants were categorised as having high adherence (≥8 points), moderate adherence (4–7 points), or low adherence (≤3 points) to the Mediterranean diet.

### 2.5. Anthropometric Measurements

Anthropometric data, including body weight, height, and waist circumference, were collected from participants following the standardised protocol of the World Health Organisation (WHO) [15]. Body weight was measured using a SECA 899 electronic scale (SECA, Hamburg, Germany), height with a SECA 217 portable stadiometer (SECA, Hamburg, Germany), and waist circumference with a SECA 201 flexible tape measure (SECA, Hamburg, Germany). Each measurement was taken three times, and the mean value was used for subsequent analyses.

Body mass index (BMI) was calculated and used to classify weight status according to the criteria of the International Obesity Task Force (IOTF) for children and the WHO classification for parents (self-reported weight and height). The waist-to-height ratio was also calculated to assess abdominal obesity in children and adolescents [19].

### 2.6. Other Parental Data

Socioeconomic factors were considered through the parents’ educational level and occupation. Their own perception of their health status was assessed, as well as the frequency of tobacco consumption.

### 2.7. Statistics

All statistical analyses were performed using IBM SPSS Statistics for Windows, version 27.0 (IBM Corp., Armonk, NY, USA). For descriptive analysis, qualitative dependent variables were expressed in frequencies and percentages, and differences between groups were analysed using the chi-square test (χ^2^). Differences with a *p* < 0.05 were considered statistically significant. Logistic regression was used to analyse the association between the grouping variable (parents’ diet quality) and the variables related to the items and total score of the KIDMED index, considering parents’ low diet quality as the reference group.

Crude and adjusted odds ratios (ORs) (age, BMI, sex, and abdominal obesity of the participant and the educational level and BMI of the parent) were calculated together with their 95% confidence intervals (CIs). The grouping variable was also associated with the sociodemographic and socioeconomic characteristics of the parents themselves, considering Parents’ Low Diet Quality as the reference group. Crude and adjusted odds ratios (ORs) (parent’s age and sex) were calculated along with their 95% confidence intervals (CIs). The *p*-values were shown, with significant differences highlighted with an asterisk (* *p* < 0.05) and highly significant differences with two asterisks (** *p* < 0.01). Pearson’s correlation was used to explore the linear association between continuous variables. The correlation coefficient (r) was calculated, and its statistical significance was evaluated using the *p*-value, considering *p* < 0.05 to be significant.

## 3. Results

Table 1 presents the sociodemographic and anthropometric characteristics of the participants according to parental diet quality in both study waves. Participants included in the analytical sample did not differ substantially from the original PASOS sample in terms of age and sex distribution. In addition, comparisons between included and excluded participants showed no significant differences in key variables, including sex, weight status, and abdominal obesity (Supplementary Table S1). In 2019–2020, no significant differences were observed between the low and high parental diet quality groups in terms of sex (female) or age group (≤12.99 years). However, differences were detected in weight status according to the IOTF BMI classification (*p* = 0.031). Children whose parents had higher diet quality showed a greater proportion of normal weight and lower prevalences of overweight, obesity, and severe obesity. In addition, abdominal obesity was significantly less frequent among children whose parents had higher diet quality (13.7% vs. 22.0%; *p* < 0.001).

In the 2022–2023 wave, no significant differences were observed between groups for sex, age group, or BMI-IOTF classification. However, abdominal obesity remained significantly less prevalent among children of parents with higher diet quality (16.8% vs. 23.9%; *p* = 0.038).

These findings suggest that parental diet quality may be associated with differences in children’s adiposity indicators, particularly abdominal obesity. To assess the robustness of this association, sensitivity analyses were conducted focusing on abdominal obesity. Analyses using parental diet quality as a continuous variable yielded consistent results (Supplementary Table S2). Similarly, analyses using tertiles of parental diet quality showed a consistent pattern, with higher parental diet quality associated with a lower likelihood of abdominal obesity.

Table 2 shows the sociodemographic characteristics of the primary caregiver according to the quality of the parental diet. In 2019–2020, differences were observed in the caregiver’s BMI (WHO criteria) between the groups (*p* = 0.042). Caregivers with high dietary quality had a higher prevalence of normal weight (54.6% vs. 44.3%) and lower proportions of pre-obesity (24.2% vs. 31.2%), and obesity. Health perception also differed in 2019–2020 (*p* = 0.025), with caregivers reporting very good health more frequently in the high diet quality group (40.0% vs. 33.1%). In both groups, however, the majority of caregivers reported good or very good health. Regular smoking differed (*p* = 0.013), being less frequent among caregivers with high diet quality (16.5% vs. 23.7%). In 2022–2023, differences in caregiver BMI remained (*p* = 0.041), with a higher prevalence of normal weight and lower levels of obesity again observed among caregivers with high dietary quality. Differences were detected in health perception (*p* = 0.003), with a higher proportion of caregivers reporting very good/good health in the high dietary quality group. Educational level showed differences in 2022–2023 (*p* < 0.001), with a higher proportion of carers with university education in the high dietary quality group (56.0% vs. 36.6%).

Table 3 shows the association between parental diet quality and that of their children. In 2019, children of parents with high dietary quality were more likely to have a high-quality diet (high KIDMED) in both the crude analysis (OR = 1.78; 95% CI: 1.38–2.30) and the adjusted analysis (OR = 1.68; 95% CI: 1.27–2.23). Conversely, a lower probability of having a low KIDMED diet was observed in the crude analysis (OR = 0.62; 95% CI: 0.39–1.00), although this association lost significance after adjustment. In 2022, these associations were maintained and even strengthened, with a higher probability of high KIDMED in children of parents with high dietary quality observed in both the crude model (OR = 1.86; 95% CI: 1.32–2.61) and the adjusted model (OR = 2.01; 95% CI: 1.35–3.00). In relation to specific dietary components, in both periods, children of parents with high dietary quality were more likely to consume cereals or bread for breakfast, eat a second daily serving of fruit, and consume fish on a regular basis. These associations were significant in both the crude and adjusted models, especially in 2022, when larger effect sizes were observed. However, daily vegetable consumption showed positive associations only in the crude analyses, losing significance after adjustment.

Table 4 shows the association between parental diet quality and sociodemographic characteristics. In 2019, parental diet quality was associated with weight status, perceived health, and certain sociodemographic factors. Normal weight was associated with a higher probability of a high-quality diet (adjusted OR = 1.66; 95% CI: 1.27–2.17), while pre-obesity was inversely associated (adjusted OR = 0.64; 95% CI: 0.48–0.86). Likewise, a very good perception of health was associated with high dietary quality (adjusted OR = 1.41; 95% CI: 1.08–1.83), while poor perception was inversely associated (adjusted OR = 0.57; 95% CI: 0.35–0.95), with poor perception being less likely in the High Diet Quality group. Parents in the High Diet Quality group were less likely to have no university education and to be unemployed. In contrast, the High Diet Quality group were more likely to be non-smokers compared to the reference group (Low Diet Quality). In 2022, normal weight was associated with a higher probability of a high-quality diet only in the crude analysis (OR = 1.52; 95% CI: 1.05–2.19), while obesity showed a consistent inverse association in both the crude model (OR = 0.33; 95% CI: 0.16–0.72) and the adjusted model (OR = 0.35; 95% CI: 0.16–0.75). A very good perception of health was strongly associated with high dietary quality (adjusted OR = 2.04; 95% CI: 1.43–2.90), while a good perception of health was inversely associated. Likewise, not having a university education was associated with a lower probability of a high-quality diet (adjusted OR = 0.48; 95% CI: 0.33–0.69), while not smoking was associated with a higher probability among parents in the High Diet Quality group (adjusted OR = 2.30; 95% CI: 1.07–4.96).

Table 5 presents the results of Pearson’s correlation analyses between parental diet quality and several dietary and anthropometric indicators in children. In 2019–2020, parental diet quality was positively correlated with children’s diet quality (KIDMED score) (r = 0.198; *p* < 0.001). Inverse correlations were also observed with children’s anthropometric indicators, although these were generally of a very small magnitude. Specifically, weak inverse correlations were found for BMI (r = −0.006; *p* = 0.036), BMI z-score (r = −0.070; *p* = 0.025), waist circumference (r = −0.068; *p* = 0.030), and waist-to-height ratio (r = −0.121; *p* < 0.001), while no significant correlation was observed with body weight.

In 2022–2023, the positive correlation between parental diet quality and children’s diet quality remained (r = 0.190; *p* < 0.001). Similarly, weak inverse correlations were observed with BMI z-score (r = −0.083; *p* = 0.048), waist circumference (r = −0.086; *p* = 0.039), and waist-to-height ratio (r = −0.091; *p* = 0.029), whereas no significant association was detected with body weight.

Overall, these results indicate that higher parental diet quality is consistently associated with better dietary quality in children across both study waves. In contrast, the correlations with anthropometric indicators were weak, suggesting that parental dietary patterns may have a clearer relationship with children’s dietary behaviours than with their anthropometric outcomes. Nevertheless, the inverse direction of the associations observed for several adiposity indicators, particularly the waist-to-height ratio, suggests that healthier parental dietary patterns may be modestly associated with more favourable body composition in children.

## 4. Discussion

The present study examined the association between parental diet quality, children’s dietary habits, and anthropometric outcomes using data from the two waves of the PASOS study (2019–2020 and 2022–2023). While previous research has suggested that parental dietary behaviours influence children’s eating patterns, this study adds to the existing evidence by demonstrating, for the first time in a large national sample, the consistency of these associations across the two independent population-based waves, while simultaneously examining parental diet quality, children’s dietary habits, and multiple anthropometric indicators, including measures of central adiposity. Overall, these findings indicate that parent–child dietary associations are stable across time and are also reflected in markers of central adiposity, reinforcing the importance of the family environment in shaping children’s dietary behaviours and related health outcomes.

Despite growing evidence on the role of the family environment in shaping children’s dietary habits, important knowledge gaps remain regarding the consistency of these associations across different population samples and the extent to which parental diet quality may relate not only to children’s dietary behaviours but also to indicators of adiposity. Few population-based studies have simultaneously examined parental diet quality, children’s dietary habits, and multiple anthropometric indicators across different time points. In this context, the current study contributes to the existing literature by demonstrating the consistency of the associations between parental diet quality and children’s dietary patterns across the two independent waves of a large national study. This approach provides novel evidence on the stability of parent–child dietary associations across time and their relationship with central adiposity in children.

With regard to anthropometric outcomes, children whose parents had higher diet quality showed more favourable indicators of body composition. In the 2019–2020 wave, children of parents with higher diet quality had a higher prevalence of normal weight, and lower prevalence of overweight and obesity. Furthermore, abdominal obesity was consistently less frequent among children whose parents had higher diet quality in both study waves. Although the correlations observed between parental diet quality and children’s anthropometric indicators were relatively weak, they were consistently negative for several markers of adiposity, including the BMI z-score, waist circumference, and waist-to-height ratio. These findings suggest that parental dietary patterns may have a modest but consistent relationship with children’s body composition.

Previous studies have reported associations between healthier dietary patterns and lower levels of adiposity in children [20,21,22]. However, much of the existing literature has focused primarily on body mass index (BMI) as the main indicator of nutritional status. The inclusion of central adiposity indicators in the present study provides additional insight into these relationships, as measures such as waist circumference and waist-to-height ratio have been increasingly recognised as sensitive markers of cardiometabolic risk in paediatric populations [23,24]. The observation that associations were more consistent for central adiposity indicators than for overall body weight suggests that parental dietary habits may influence body fat distribution rather than body weight alone.

The study also identified differences in caregivers’ characteristics according to parental diet quality. Parents with higher diet quality were more likely to have normal weight, report better perceived health, and present healthier lifestyle behaviours. In addition, differences were observed in socioeconomic characteristics, particularly in educational attainment. These findings are consistent with previous literature showing that healthier dietary patterns tend to cluster with other health-promoting behaviours and favourable socioeconomic conditions [25,26]. Higher educational attainment may facilitate healthier dietary choices through greater nutritional knowledge, improved health literacy, and increased access to healthier foods and lifestyle opportunities [27,28]. These factors may also influence the home food environment, which plays a key role in shaping children’s dietary habits.

One of the most relevant findings of this study was the strong association between parental diet quality and children’s dietary habits. In both study waves, children of parents with higher diet quality were significantly more likely to follow healthier dietary patterns, as reflected by higher KIDMED scores. In 2019, children of parents with higher diet quality had a higher probability of high adherence to the Mediterranean diet (adjusted OR = 1.68), and this association was even stronger in 2022 (adjusted OR = 2.01). These results are consistent with previous studies highlighting the influence of parental dietary behaviours on children’s eating habits [13,29].

Contrary to some previous studies suggesting sex-related differences in dietary behaviours among adolescents [30], no clear sex differences were observed in the current study. This may be explained by the relatively narrow age range of the sample, as sex-related differences in dietary behaviours may become more pronounced during mid-to-late adolescence. Moreover, cultural and environmental factors, as well as the specific dietary assessment tools used, may influence the detection of such differences. These findings suggest that sex-related disparities in dietary patterns may be less evident at earlier developmental stages, particularly within the context of shared family environments [31].

Parents play a central role in shaping children’s dietary behaviours through several mechanisms, including modelling of food choices, determining food availability at home, and structuring family meals [32,33,34]. Children tend to imitate parental eating behaviours and are more likely to adopt similar dietary patterns when healthy foods are regularly consumed within the household. In addition, parental dietary habits influence the types of foods that are purchased, prepared, and served at home, which directly affects children’s food exposure and preferences.

Beyond overall dietary patterns, several specific dietary behaviours were associated with parental diet quality. Children of parents with higher diet quality were more likely to consume cereals or bread at breakfast, eat a second daily serving of fruit, and regularly consume fish. These findings align with previous studies indicating that children’s consumption of healthy foods is strongly influenced by parental dietary habits and by the broader home food environment [13,29]. Parental adherence to healthy dietary patterns, such as the Mediterranean diet, may facilitate the development of similar dietary habits among children by promoting healthier meal routines and improving the availability of nutritious foods at home [27,35].

Finally, correlation analyses showed consistent associations between parental diet quality and children’s dietary and anthropometric indicators. In both study waves, parental diet quality was positively correlated with children’s diet quality, suggesting that parents with healthier dietary habits tend to have children with healthier eating patterns. In contrast, correlations with anthropometric indicators were weaker, although consistently negative for several markers of adiposity. This pattern suggests that parental dietary habits may influence children’s dietary behaviours more directly than their anthropometric outcomes. Such findings are consistent with the multifactorial nature of childhood obesity, which is influenced not only by dietary habits but also by factors such as physical activity, genetic predisposition, and broader environmental influences [20,21,22,36,37,38].

Taken together, these findings highlight the central role of the family environment in shaping children’s dietary behaviours and, to a lesser extent, their anthropometric outcomes. The consistency of the associations observed across two independent waves of the PASOS study strengthens the evidence supporting the influence of parental dietary patterns on children’s health-related behaviours. From a public health perspective, these results reinforce the importance of family-based strategies aimed at promoting healthy eating habits and preventing childhood obesity. Interventions targeting the household environment and encouraging healthier dietary behaviours among parents may contribute to improving children’s dietary patterns and supporting healthier growth trajectories. Overall, these findings provide robust population-based evidence supporting the role of parental diet quality as a key determinant of children’s dietary behaviours and related health outcomes.

### Strengths and Limitations

This study has several strengths. First, despite some missing data, the study still includes a large and well-characterised paediatric population with data collected at two different time points, allowing the assessment of the consistency of associations over time. Second, the use of validated dietary quality indices for both parents and children strengthens the reliability of the dietary assessment. Third, the inclusion of multiple anthropometric indicators, particularly measures of central adiposity, provides a more comprehensive evaluation of body composition beyond BMI alone. Additionally, the consideration of parental sociodemographic and lifestyle factors allowed for a more holistic understanding of the family context influencing children’s diet and health.

Nevertheless, some limitations should be acknowledged. The observational design precludes causal inference, and residual confounding cannot be completely ruled out despite adjustment for relevant covariates. Dietary intake was self-reported, which may be subject to recall bias and social desirability bias, particularly among parents. In addition, as several variables were obtained through self-reported questionnaires, the possibility of common method bias cannot be completely excluded; however, this risk may be partially mitigated by the use of validated instruments and standardised data collection procedures within the PASOS study. Furthermore, differences in the number of participants across the study waves and missing data may have introduced some degree of selection bias and could potentially affect the representativeness of the analytical sample. However, comparisons between included and excluded participants did not show substantial differences in key variables, supporting the robustness of the analytical sample. Although associations were consistent, the effect sizes were modest, which may reflect the multifactorial nature of childhood obesity and dietary behaviours. Finally, information on other potentially relevant factors, such as detailed parental feeding styles or the broader food environment outside the home, was not available and should be considered in future research.

## 5. Conclusions

Higher parental diet quality was consistently associated with healthier dietary patterns in children, while its relationship with anthropometric indicators appeared to be more modest. These findings highlight the importance of the family environment in shaping children’s eating behaviours and suggest that interventions aimed at improving parental dietary habits may contribute to healthier dietary patterns in children. Accordingly, strategies targeting the family context, rather than children alone, may be particularly relevant for improving diet quality and preventing childhood obesity. Future research should prioritise longitudinal and intervention studies to clarify the causal pathways linking parental dietary patterns to children’s health outcomes and to better understand the role of parental feeding practices, food availability, and broader socio-environmental factors.

## Figures and Tables

**Table 1:** Sociodemographic characteristics of participants according to the quality of their parents’ diet.

	Parental Low Diet Quality ^§^	Parents High Diet Quality ^§^	*p*-Value ^‡^
Female (*n*; %)			
2019–2020	285 (49.7)	225 (49.6)	0.977
2022–2023	134 (41.6)	114 (45.6)	0.340
Children-age ≤ 12.99 years (*n*; %)			
2019–2020	357 (62.2)	268 (59.0)	0.302
2022–2023	226 (70.2)	166 (66.4)	0.333
BMI IOTF (*n*; %)			
2019–2020			0.031
Underweight	37 (6.3)	22 (4.9)	
Normal weight	376 (65.5)	332 (73.1)	
Overweight	122 (21.3)	81 (17.8)	
Obesity	28 (4.9)	16 (3.5)	
Severe obesity	9 (1.6)	1 (0.2)	
2022–2023			0.284
Underweight	17 (5.3)	19 (7.6)	
Normal weight	205 (63.7)	175 (70.0)	
Overweight	76 (23.6)	44 (17.6)	
Obesity	18 (5.6)	9 (3.6)	
Severe obesity	6 (1.9)	3 (1.2)	
Abdominal obesity (*n*; %)			
2019–2020			<0.001
No	446 (77.7)	390 (85.9)	
Yes	126 (22.0)	62 (13.7)	
2022–2023			0.038
No	245 (76.1)	208 (83.2)	
Yes	77 (23.9)	42 (16.8)	

Abbreviations: BMI: body mass index. ^§^ Parents’ diet quality was categorised using the sample median (50th percentile; score = 39) as the cut-off point: ≤p50 indicates low diet quality and >p50 indicates high diet quality. ^‡^ Differences in prevalence across groups were examined using χ^2^. The number of observations may vary across variables due to missing responses in questionnaire items.

**Table 2:** Sociodemographic characteristics of the primary caregiver according to their own diet quality.

	Parents Low Diet Quality ^§^	Parents High Diet Quality ^§^	*p*-Value ^‡^
BMI WHO (*n*; %)			
2019–2020			0.042
Underweight	12 (2.0)	11 (2.4)	
Normal weight	254 (44.3)	248 (54.6)	
Pre-obesity	179 (31.2)	110 (24.2)	
Obesity class I	58 (10.1)	38 (8.4)	
Obesity class II	14 (2.4)	5 (1.1)	
Obesity class III	3 (0.5)	2 (0.4)	
2022–2023			0.041
Underweight	8 (2.5)	7 (2.8)	
Normal weight	149 (46.3)	137 (54.8)	
Pre-obesity	89 (27.6)	62 (24.8)	
Obesity class I	27 (8.4)	6 (2.4)	
Obesity class II	4 (1.2)	2 (0.8)	
Obesity class III	1 (0.3)	1 (0.4)	
Health perception (*n*; %)			
2019–2020			0.025
Very good	190 (33.1)	182 (40.0)	
Good	309 (53.8)	226 (49.8)	
Not good	50 (8.7)	26 (5.7)	
2022–2023			0.003
Very good	117 (36.3)	131 (52.4)	
Good	154 (47.8)	91 (36.4)	
Not good	23 (7.1)	11 (4.4)	
Educational level (*n*; %)			
2019–2020			0.088
High level	202 (35.2)	189 (41.6)	
Intermediate level	295 (51.4)	195 (42.9)	
Low level or not	52 (9.0)	52 (11.5)	
2022–2023			<0.001
High level	118 (36.6)	140 (56.0)	
Intermediate level	152 (47.2)	80 (32.0)	
Low level or not	24 (7.4)	13 (5.2)	
Occupation (*n*; %)			
2019–2020			0.172
Working	491 (85.6)	408 (89.9)	
Not working	59 (10.3)	28 (6.1)	
2022–2023			0.746
Working	269 (83.6)	217 (86.8)	
Not working	27 (8.3)	16 (6.4)	
Smoking status (*n*; %)			
2019–2020			0.013
Current smoker	136 (23.7)	75 (16.5)	
Former smoker	140 (24.4)	143 (31.5)	
Never smoker	273 (47.6)	218 (48.0)	
2022–2023			0.202
Current smoker	27 (8.4)	12 (4.8)	
Former smoker	32 (10.0)	33 (13.2)	
Never smoker	58 (18.0)	53 (21.2)	

Abbreviations: BMI: body mass index. ^§^ Parents’ diet quality was categorised using the sample median (50th percentile; score = 39) as the cut-off point: ≤p50 indicates low diet quality and >p50 indicates high diet quality. ^‡^ Differences between low and high parental diet quality groups were assessed using the χ^2^ test. The number of observations may vary across variables due to missing responses in some caregiver questionnaire items.

**Table 3:** Association between the quality of the diet of parents and that of their children.

		Parents High Diet Quality ^§^ (2019)	Parents High Diet Quality ^§^ (2022)
KIDMED Index			
Low	Crude OR	0.62 (0.39–1.00) *	0.54 (0.29–1.01)
	Adjusted OR	0.69 (0.42–1.15)	0.57 (0.28–1.17)
Medium	Crude OR	0.65 (0.50–0.84) **	0.66 (0.47–0.93) *
	Adjusted OR	0.67 (0.51–0.89) *	0.61 (0.42–0.90) *
High	Crude OR	1.78 (1.38–2.30) **	1.86 (1.32–2.61) **
	Adjusted OR	1.68 (1.27–2.23) **	2.01 (1.35–3.00) **
Q3. Has cereals or grains (bread., etc.) for breakfast (+)	Crude OR	1.41 (1.08–1.84) **	1.45 (1.03–2.03) *
	Adjusted OR	1.34 (1.00–1.78) *	1.53 (1.04–2.24) *
Q6. Takes a second serving of fruit daily (+)	Crude OR	1.58 (1.22–2.03) **	1.56 (1.11–2.19) **
	Adjusted OR	1.61 (1.21–2.13) **	1.43 (0.97–2.12)
Q8. Consumes raw or cooked vegetables daily (+)	Crude OR	1.34 (1.03–1.75) **	1.44 (1.01–2.07) *
	Adjusted OR	1.26 (0.94–1.68)	1.31 (0.87–1.98)
Q10. Regular fish consumption (at least 2–3 week) (+)	Crude OR	1.41 (1.07–1.84) **	1.91 (1.35–2.71) **
	Adjusted OR	1.42 (1.05–1.90) *	2.15 (1.44–3.20) **

Abbreviations: CI: confidence interval. KidMed: OR: odds ratio. ^§^ Parents’ diet quality was categorised using the sample median (50th percentile; score = 39) as the cut-off point: ≤p50 indicates low diet quality and >p50 indicates high diet quality. Adjusted OR: adjusted by age kid, BMI kid, gender kid, abdominal obesity kid, level education parents, and BMI parents. * *p*-value < 0.05. ** *p*-value < 0.01. Considering Parents’ Low Diet Quality as the reference group.

**Table 4:** Relationship between the quality of parents’ diets and their own sociodemographic/socioeconomic characteristics.

		Parents High Diet Quality ^§^ (2019)	Parents High Diet Quality ^§^ (2022)
BMI WHO			
Underweight	Crude OR	1.15 (0.50–2.64)	1.13 (0.40–3.18)
	Adjusted OR	1.20 (0.52–2.76)	1.17 (0.41–3.36)
Normal weight	Crude OR	1.56 (1.20–2.03) **	1.52 (1.05–2.19) *
	Adjusted OR	1.66 (1.27–2.17) **	1.45 (0.99–2.10)
Pre-obesity	Crude OR	0.69 (0.52–0.91) **	0.86 (0.58–1.27)
	Adjusted OR	0.64 (0.48–0.86) **	0.90 (0.61–1.34)
Obesity	Crude OR	0.72 (0.49–1.07)	0.33 (0.16–0.72) **
	Adjusted OR	0.72 (0.48–1.07)	0.35 (0.16–0.75) **
Health Perception			
Very Good	Crude OR	1.36 (1.05–1.77) *	1.94 (1.37–2.75) **
	Adjusted OR	1.41 (1.08–1.83) *	2.04 (1.43–2.90) **
Good	Crude OR	0.84 (0.65–1.09)	0.58 (0.41–0.82) **
	Adjusted OR	0.84 (0.65–1.08)	0.56 (0.39–0.80) **
Not Good	Crude OR	0.63 (0.39–1.04)	0.58 (0.28–1.22)
	Adjusted OR	0.57 (0.35–0.95) *	0.57 (0.27–1.21)
University degree (−)	Crude OR	0.76 (0.59–0.98) *	0.44 (0.31–0.63) **
	Adjusted OR	0.81 (0.62–1.06)	0.48 (0.33–0.69) **
Working (−)	Crude OR	0.57 (0.36–0.91) *	0.73 (0.38–1.40)
	Adjusted OR	0.55 (0.34–0.87) *	0.79 (0.41–1.51)
Smoking (−)	Crude OR	1.58 (1.15–2.17) **	2.15 (1.02–4.51) *
	Adjusted OR	1.53 (1.11–2.10) **	2.30 (1.07–4.96) *

Abbreviations: CI: confidence interval. KidMed: OR: odds ratio. ^§^ Parents’ diet quality was categorised using the sample median (50th percentile; score = 39) as the cut-off point: ≤p50 indicates low diet quality and >p50 indicates high diet quality.Adjusted OR: Adjusted by age and gender of parents. * *p*-value <0.05. ** *p*-value <0.01. Considering Parents’ Low Diet Quality as the reference group.

**Table 5:** Pearson correlations between parental diet quality and children’s dietary quality and anthropometric indicators in the 2019–2020 and 2022–2023 PASOS waves.

	r (*p*-Value)
2019	
Quality of children diet (KidMed score)	0.198 (<0.001)
Weight (kg)	−0.009 (0.764)
BMI (kg/m^2^)	−0.006 (0.036)
Standarized BMI Score (z score)	−0.070 (0.025)
Waist circumference (mean in cm)	−0.068 (0.030)
Ratio/coefficient between waist circumference (cm) and height (cm)	−0.121 (<0.001)
2022	
Quality of children diet (KidMed score)	0.190 (<0.001)
Weight (Kg)	−0.045 (0.284)
BMI (kg/m^2^)	−0.073 (0.080)
Standarized BMI Score (z score)	−0.083 (0.048)
Waist circumference (mean in cm)	−0.086 (0.039)
Ratio/coefficient between waist circumference (cm) and height (cm)	−0.091 (0.029)

Abbreviations: BMI: body mass index; cm: centimetres (UK); kg: kilograms; KidMed: Mediterranean diet quality index; m^2^: square metres. Pearson correlation coefficients (r) and corresponding *p*-values are shown for the association between parental diet quality and children’s dietary and anthropometric indicators. Positive values indicate higher child diet quality with higher parental diet quality, whereas negative values indicate lower anthropometric indicators with higher parental diet quality. Statistical significance was set at *p* < 0.05.

## Data Availability

There are restrictions on the availability of data for this trial due to the signed consent agreements around data sharing, which only allow for access to external researchers for studies following the project purposes. Requestors wishing to access the trial data used in this study can make a request to pep.tur@uib.es.

## Supplementary Materials

The following supporting information can be downloaded at: https://www.mdpi.com/article/10.3390/nu18071038/s1, Table S1: Comparison of included vs excluded participants by study wave; Table S2: Association between parental diet quality and abdominal obesity in children.

## Abbreviations

BMI	Body mass index
KIDMED	Mediterranean diet quality index
OR	Odds ratio
SD	Standard deviations
SDQS	Short diet quality screener
WHO	World Health Organisation
