# Supplementary material for: Family Eating Habits and Dietary Quality of Spanish Children and Adolescents: The PASOS Study
Source: Nutrients. 2026 Mar 25;18(7):1038. doi: 10.3390/nu18071038 (PMC13074208; doi:10.3390/nu18071038)
Supplement: Supplementary file 1 [file nutrients-18-01038-s001.zip › nutrients-4195461-supplementary.pdf]

**Supplementary Materials**

**Table S1.** Comparison of included vs excluded participants by study wave.

| | | **2019–2020 Excluded (%)** | **2019–2020 Included (%)** | **p-value** | **2022–2023 Excluded (%)** | **2022–2023 Included (%)** | **p-value** |
|---|---|---|---|---|---|---|---|
| Sex | Male | 50.7 | 48.2 | 0.209 | 48.5 | 49.6 | 0.595 |
| | Female | 49.3 | 51.8 | | 51.5 | 50.4 | |
| Weight Status (BMI IOTF) | Underweight | 5.5 | 5.2 | 0.728 | 6.5 | 6.2 | 0.072 |
| | Normal weight | 63.0 | 64.8 | | 64.3 | 65.9 | |
| | Overweight | 22.7 | 22.4 | | 20.9 | 21.0 | |
| | Obesity | 6.8 | 6.2 | | 5.8 | 5.8 | |
| | Severe obesity | 2.0 | 1.4 | | 2.4 | 1.0 | |
| Abdominal Obesity | No | 76.9 | 77.4 | 0.783 | 78.3 | 80.9 | 0.121 |
| | Yes | 23.1 | 22.6 | | 21.7 | 19.1 | |

Percentages are column percentages. P-values were obtained using chi-square tests comparing included and excluded participants within each study wave.

**Table S2.** Association between parental diet quality and abdominal obesity in children

| Parental diet quality | **OR (CI 95%)** | **P-value** |
|---|---|---|
| SDQS continuous | 0.910 (0.875-0.948) | <0.001 |
| SDQS tertiles | | |
| Middle vs lowest | 0.662 (0.484-0.904) | 0.010 |
| Highest vs lowest | 0.514 (0.378-0.698) | <0.001 |

Odds ratios (Exp(B)) and 95% confidence intervals (CI) were obtained from logistic regression models. The lowest tertile of parental diet quality was used as the reference category. Models included parental diet quality as either a continuous variable or tertiles.
